# Polyglycerol‐Based Biomedical Matrix for Immunomagnetic Circulating Tumor Cell Isolation and Their Expansion into Tumor Spheroids for Drug Screening

**DOI:** 10.1002/adhm.202300842

**Published:** 2023-07-21

**Authors:** Peng Tang, Boonya Thongrom, Smriti Arora, Rainer Haag

**Affiliations:** ^1^ Institute for Chemistry and Biochemistry Freie Universität Berlin Takustr. 3 14195 Berlin Germany

**Keywords:** 3D hydrogels, biomaterials, circulating tumor cells, drug screening, multicellular tumor spheroids, polyglycerol biointerfaces

## Abstract

Circulating tumor cells (CTCs) are established as distinct cancer biomarkers for diagnosis, as preclinical models, and therapeutic targets. Their use as preclinical models is limited owing to low purity after isolation and the lack of effective techniques to create 3D cultures that accurately mimic in vivo conditions. Herein, a two‐component system for detecting, isolating, and expanding CTCs to generate multicellular tumor spheroids that mimic the physiology and microenvironment of the diseased organ is proposed. First, an antifouling biointerface on magnetic beads is fabricated by adding a bioinert polymer layer and conjugation of biospecific ligands to isolate cancer cells, dramatically enhancing the selectivity and purity of the isolated cancer cells. Next, the isolated cells are encapsulated into self‐degradable hydrogels synthesized using a thiol‐click approach. The hydrogels are mechanochemically tuned to enable tumor spheroid growth to a size greater than 300 µm and to further release the grown spheroids while retaining their tumor‐like characteristics. In addition, drug treatment highlights the need for 3D culture environments rather than conventional 2D culture. The designed biomedical matrix shows potential as a universal method to ensure mimicry of in vivo tumor characteristics in individual patients and to improve the predictability of preclinical screening of personalized therapeutics.

## Graphical abstract

A straightforward and effective two‐component system for the capture, 3D spheroid growth of circulating tumor cells and subsequent implementation for drug screening is presented. The technique provides the next step for personalized therapeutic management through connecting liquid biopsy, oncological research, and cancer treatment.

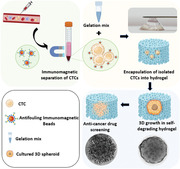

## 1 Introduction

Cancer metastasis is a multistep process involving dynamic changes in mutational and phenotypic landscapes.^[^ ^1^ ^]^ These properties necessitate the constant monitoring of cancer patients to provide the most efficient care. However, the readily available primary tumor biopsies do not reflect the wide heterogeneity of tumors; in fact, they may only reveal signatures specific to local tumors, lacking the aberrant genomic changes that occur during the metastatic course. Thus, it becomes extremely challenging to devise a common treatment strategy to target different metastatic populations.^[^ ^2^ ^]^ To achieve favorable long‐term clinical outcomes, it is imperative to establish focused therapeutic treatments that are customized to each patient.^[^ ^3^ ^]^ In this context, circulating tumor cells (CTCs) have been clinically validated as the first real‐time, cellular, liquid biopsy cancer biomarkers that could enable highly precise and personalized cancer treatment.^[^ ^4^ ^]^ Isolating and expanding CTCs from distinct treatment stages may therefore illuminate the evolution of specific tumor characteristics that can guide therapeutic decisions.^[^ ^5^ ^]^

Numerous strategies have been developed to isolate and culture CTCs among a billion normal blood cells, taking advantage of their unique biological and physical properties. Existing CTC isolation techniques include functionalized nanostructured surfaces based on cell‐substrate affinity,^[^ ^6^ ^]^ microfluidic devices that promote cell–surface contact,^[^ ^7^ ^]^ immunomagnetic beads immobilized with capture biomolecules,^[^ ^8^ ^]^ and microfilter devices for isolating tumor cells based on their varying size.^[^ ^9^ ^,4b]^ Furthermore, these methods can be integrated with 3D expansion models, like spheroids, organoids, and xenografts, that are becoming increasingly vital for high‐throughput drug screening and for studying metastases.^[^ ^10^ ^]^ Such systems have clinical applicability to human malignancies as they can accurately replicate the key elements of the 3D architecture of the tumor microenvironment that results from altered cellular morphology, motility, and polarity. Although the personalized patient‐derived xenograft is a very promising model, this technique is typically expensive, low‐throughput, and very challenging to scale up.^[^ ^11^ ^]^ These challenges have inspired the transition to 3D gel‐based matrices like collagen and Matrigel^[^ ^12^ ^]^ to better reflect the functional pathophysiology of in vivo tumors and simulate the interactions between cells and the extracellular matrix (ECM). These matrices comprise a crosslinked polymer network that can be adjusted to customize the stiffness and viscoelasticity of soft tissues.^[^ ^13^ ^]^ In addition, hydrogels can offer a wide range of biochemical and biophysical cues for in vitro cell growth by using natural ECM molecules or synthetic materials modified by cell adhesion receptors, making them suitable for the challenge of culturing primary cancer cells.^[11a,14]^ Matrigel‐based and synthetic hydrogels have been used for expanding CTCs from cancer patients while preserving the expression of epithelial‐cell adhesion molecule (EpCAM) and β‐catenin.^[^ ^15^ ^]^ However, the advantage of using synthetic hydrogels is the flexibility to include desired levels of modifications along biological or physical parameters, such as biodegradability, porosity, growth factors, and cleavage sites. A combinatorial two‐step approach has been proposed by Liao et al., integrating a negative‐selection CTC isolation and subsequent spheroid cell culture to test for potential cancer metastasis and thus the prognosis for disease.^[^ ^16^ ^]^ However, negative CTC isolation techniques often have lower capture selectivity, purity, and viability, limitations which could restrict their use for cell proliferation and downstream analysis at clinical levels.

**Figure 1:**
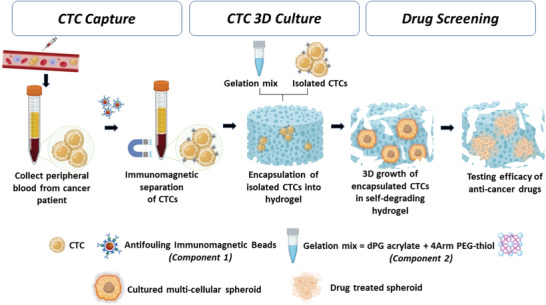
Schematic illustration of the polyglycerol‐based biomatrix designed for detecting, isolating, and expanding CTCs from cancer patients' blood to monitor early‐stage cancer and determine the potency and efficacy of anticancer drugs for individual cancer patients.

To address these issues, we present a two‐component system that can detect, isolate, and culture CTC tumor spheroids from cancer patients’ blood at various treatment stages. This system could be used to monitor early‐stage cancer and assess the potency and efficacy of anticancer drugs for individual cancer patients (**Figure** **1**). The first component involves immunomagnetic beads fabricated with dendritic polyglycerol (dPG)‐catechol, an anti‐biofouling coating, and functionalized with anti‐EpCAM antibodies to isolate EpCAM‐overexpressing CTCs (in this case, MCF‐7) with high efficiency, selectivity, and viability. The dPG‐based polymeric coating acts as a potent antifouling backdrop to fend off nonspecific blood cells’ and proteins’ adherence (red blood cells, white blood cells, proteins, etc.). These immunomagnetically isolated cells were embedded inside a synthetic degradable hydrogel composed of dPG and four‐arm polyethylene glycol (PEG) to grow 3D spheroids. By simply adjusting the concentration and the ratio of two gel components, an optimal ECM‐mimicking environment can be provided to grow multicellular tumor spheroids (MCTSs). Such a 3D matrix facilitates better intercellular communication and encourages self‐assembly to generate structures that better resemble in vivo organization. Furthermore, the cultured tumor spheroids in 3D were screened for dose‐dependent effects with the chemotherapeutic drug candidates doxorubicin and paclitaxel.

## 2 Results and Discussion

### 2.1 Antifouling Immunomagnetic Beads

**Figure 2:**
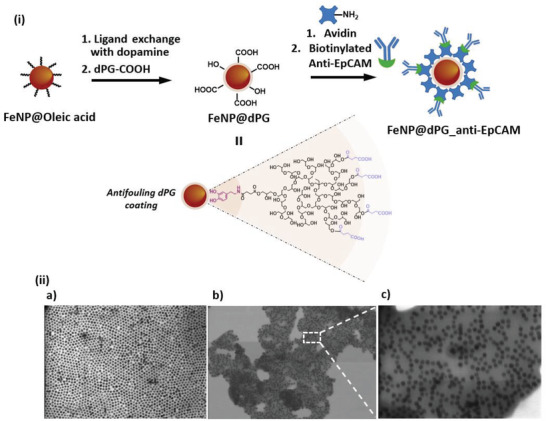
i) Schematic representation of the synthesis of anti‐EpCAM antibody‐functionalized dPG‐coated FeNPs; ii) SEM images of FeNPs with an average particle size of ≈20 nm and different surface coatings: a) oleic acid coating on FeNPs. Scale = 200 nm, b,c) dPG coating on FeNPs. Scale = 250 nm, and the corresponding zoomed‐in image, Scale = 100 nm.

dPG is considered an excellent alternative to PEG for its extraordinary performance and functionality. It has been used as a bioinert and biocompatible surface coating polymer, as described by earlier studies from our group.^[^ ^17^ ^]^ Now, we fabricate magnetic iron nanoparticles (FeNPs) with a multifunctional dPG coating (**Figure** **2**i). First we synthesized homogenous magnetic nanoparticles of ≈20 nm in size, functionalized with oleic acid using a known protocol.^[^ ^18^ ^]^ Furthermore, oleic acid was replaced with dopamine to add water solubility and reactive amino groups. Then, the amino groups were coupled with succinic acid‐functionalized dPG (dPG‐SA). The average molecular weight of the dPG coating polymer is about 19 kDa, with 60% acidic functionalities, and an estimated 81 groups of succinic acid per dPG molecule as quantified by ^1^H NMR end‐group analysis. Transmission electron microscopy (TEM) imagery clearly depicts the change of the patterned arrangement of the particles to a polymeric coating when the ligand is switched from oleic acid to dPG (Figure 2ii). Here, the catechol groups and their oxidized derivatives serve as an anchoring domain that forms coordinate bonds with the FeNP surface to secure the coating of the dPG layer on the FeNPs.^[^ ^19^ ^]^ The dPG backbone, once hydrated by the water molecule, acts as a hydrophilic domain that can significantly inhibit cell adhesion and the absorption of nonspecific proteins.^[^ ^20^ ^]^ This strong bioinert scaffold helps prevent blood cells and non‐specific protein absorption from interfering with CTC isolation. A prominent CTC biomarker is EpCAM (epithelial cell adhesion molecule), which is overexpressed in most adenocarcinoma CTCs but negatively expressed in healthy blood cells.^[^ ^21^ ^]^ As a result, the bioactive anti‐EpCAM antibody‐based CTC separation approaches have been used extensively.^[^ ^22^ ^]^ Hence, to specifically capture CTCs, we used biotin ligand to further immobilize the biospecific anti‐EpCAM antibody onto the dPG coating. This functionalization was performed using an avidin biolinker and was based on the non‐covalent interaction between biotin and avidin, which is the one of the strongest noncovalent interactions in nature (the dissociation constant of avidin and biotin is 10^−15^ m).^[^ ^23^ ^]^ In addition, since biotin is a small molecule and is biorthogonal to the conjugated antibodies, it does not impair the bioactivity of the conjugated antibodies.^[^ ^24^ ^]^ Along with its antifouling properties and hydrophilicity, the multivalent nature of dPG allows high antibody functionalization. The resulting biointerfaces are termed as FeNP@dPG_anti‐EpCAM.

### 2.2 Cell Capture Efficiency of the Antifouling Immunomagnetic Beads

**Figure 3:**
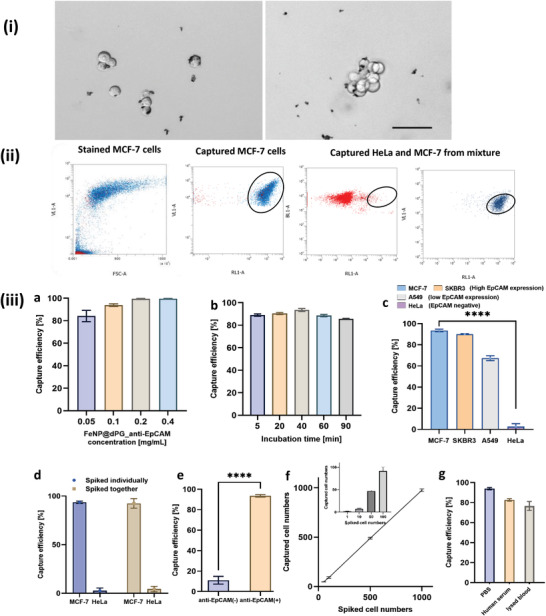
i) Bright‐field images of single and clustered MCF‐7 cells captured using anti‐EpCAM antibody‐modified dPG‐coated FeNPs (FeNP@dPG_anti‐EpCAM). Scale bar = 50 µm; ii) representative flow cytometry images for stained MCF‐7 cells before and after capture and a mixture of MCF‐7 and HeLa cells. The circled population indicates where the cells are using FeNP@dPG_anti‐EpCAM; iii) representative capture efficiency (determined by flow cytometry) using: a) different concentrations of FeNP@dPG_anti‐EpCAM particles (0.05, 0.1, 0.2, and 0.4 mg mL^−1^), capture time = 40 min; b) different incubation times (5, 20, 40, 60, and 90 min) with 0.1 mg mL^−1^ of FeNP@dPG_anti‐EpCAM particles; c) different cell lines (MCF‐7, SKBR3, A549, HeLa) with different EpCAM expression; d) different cancer cell lines with varied EpCAM expressions (MCF‐7 (EpCAM^+^), HeLa (EpCAM^−^); MCF‐7 cells mixed with HeLa cells in serum‐free cell culture medium), capture time = 40 min; e) different particles (FeNP@dPG and FeNP@dPG_anti‐EpCAM at 0.1 mg mL^−1^), capture time = 40 min; f) different numbers of spiked MCF‐7 cells (100, 500, and 1000 cells mL^−1^) in cell culture media, capture time = 40 min. The inset indicates the capture performance for low concentrations of cancer cells (1, 10, 50, and 100 cells mL^−1^) spiked in cell culture media. g) Different incubation media (DPBS, human serum, lysed blood), capture time = 40 min; data are presented as mean ± standard deviation, *n* = 3. Statistical analysis was performed by using a one‐way ANOVA test. *p < 0.05; **p < 0.01; ***p < 0.001; ****p < 0.0001. N.S. denotes not significant at *p* > 0.05.

Immunomagnetic iron nanoparticles equipped with anti‐EpCAM antibodies can specifically recognize and capture EpCAM^+^ cancer cells. In this study, the MCF‐7 cell line was used as the model cancer cell line, while the HeLa cell line, which is EpCAM^−^, was used as the negative control. The capture efficiencies under different conditions were investigated using flow cytometry. MCF‐7 cells were marked with violet fluorescent dye, genetically modified HeLa cells expressed green fluorescent protein, and the iron nanoparticles were conjugated with red fluorescent marker. The captured cells would be isolated by magnetic separation and would show both violet/red fluorescence for MCF‐7 cells and green/red fluorescence for HeLa cells, respectively. In **Figure** **3**i, microscopic images show the binding of iron nanoparticles with cells, and it is assumed that the iron nanoparticles could result in aggregation of cells.^[8b]^

To quantify the capture efficiency, we used flow cytometry, which is demonstrated in Figure 3ii. The population of the captured MCF‐7 cells is represented with high violet (stained cancer cells) and red fluorescence (stained immunomagnetic beads). The capture methodology was optimized by investigating the concentration of immunomagnetic beads (FeNP@dPG_anti‐EpCAM) and their incubation time at 100 000 cells mL^−1^. As the concentration of the immunomagnetic beads rose from 0.05 to 0.4 mg mL^−1^, capture efficiency increased from 84.2% to 99.6% (Figure 3iii a). Higher immunomagnetic bead concentrations, however, can result in severe aggregation, which could squander particles and further influence the hydrogel behavior in the subsequent cell‐encapsulated hydrogel formation, ultimately impairing future cell growth. Even though the antibody/antigen contact happens extremely quickly, a longer incubation period with gentle mechanical mixing promotes the conjugation of cells with iron nanoparticles. Around 89.0% of capture efficiency was already attained after only 5 min of incubation, increasing to 93.6% after 40 min. After 90 min, however, the capture efficiency decreased to 85.4% (Figure 3iii b). This decline could be related to the loss of cell activity after longer periods of mixing in Dulbecco's buffered saline (DPBS) solution. As a result, 0.1 mg mL^−1^ of FeNP@dPG_anti‐EpCAM and 40 min of incubation were used as a standard condition for MCF‐7 cell capture, with 93.6% capture efficiency. As mentioned above, the specific recognition and capture were done by anti‐EpCAM. High EpCAM expressing cell lines, MCF‐7 and SKBR3, were captured with efficiency of 93.56% and 90.04%, respectively. The low EpCAM expressing cell line, A549 was captured with 67.304% efficiency while the negative EpCAM expressing cell line was captured with an efficiency of 2.63% (Figure 3iii c). When we attempted to individually capture HeLa cells with FeNP@dPG_anti‐EpCAM, only 2.6% capture efficiency was reached, rising to 4.3% when spiked together with MCF‐7 cells; in this case, the capture efficiency of MCF‐7 remained at 92.4% (Figure 3iii d). Furthermore, the specificity was proven by FeNP@dPG without anti‐EpCAM, with only 11.1% capture efficiency in comparison with 93.6% achieved with FeNP@dPG_anti‐EpCAM (Figure 3iii e). The dPG coating layer on the outer shell of nanoparticles also prevented nonspecific binding with cells or proteins. The capture sensitivity of immunomagnetic beads was identified by spiking cells from as low as 1 to 1000 cells mL^−1^ (Figure 3iii f). The capture efficiency remained around 90% at all spiked cell counts. In order to mimic clinical conditions, we spiked cancer cells in DPBS, human serum, and healthy human blood, respectively. In serum, cancer cells were captured with 82.7% efficiency, and in healthy human blood, it was found to remain at 76.6% (Figure 3iii g). The presence of different proteins, cells, and other blood components had an impact on the capture efficiency to a great extent. Although capture efficiency decreased slightly, it is still viable for 3D culture. The outstanding antifouling property of the dPG layer and the specificity of anti‐EpCAM validates the high performance of FeNP@dPG_anti‐EpCAM in capturing EpCAM^+^ cancer cells like MCF‐7 cells. Besides, the easy separation of the cells using immunomagnetic iron nanoparticles facilitates their direct encapsulation in hydrogels.

### 2.3 Synthesis of Gel Precursors, Hydrogel Formation, and Rheological Characterization

The follow‐up tumor spheroid formation demands a simple and fast hydrogel matrix in aqueous solution with no release of toxic by‐products. In light of these requirements, thiol‐click chemistry is preferred because it produces no by‐products and reacts quickly at physiological pH. The resulting hydrogel is also self‐degradable, making it possible to extract formed spheroids for further morphological analysis and anticancer drug testing. Among olefinic acceptors, acrylate has proven to be a good candidate for thiol‐click coupling to fabricate hydrogel, which can be gradually degraded when incubated at 37 °C.^[^ ^25^ ^]^ Herein, we used dPG and four‐arm polyethylene glycol (four‐arm PEG) as the precursors for gelation. dPG, employed as mentioned as a coating polymer on FeNPs, was also used as the main component in hydrogel formation, owing to its efficient functionality and bioinertness.

**Figure 4:**
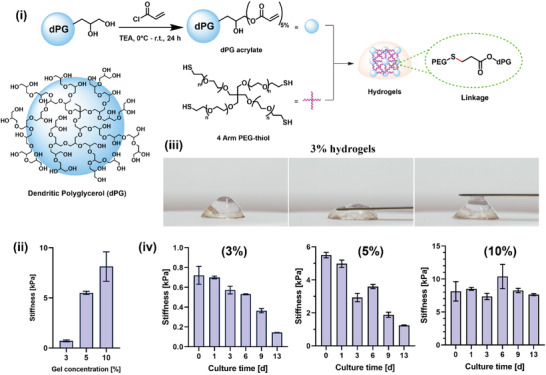
i) Synthesis of dPG‐acrylate, followed by hydrogel fabrication between dPG‐acrylate and four‐arm PEG‐thiol, for expansion of immunomagnetically isolated MCF‐7 cells into tumor spheroids. Idealized structure of dendritic polyglycerol (dPG); ii) representative change in stiffness (kPa) with variation in hydrogel concentration; iii) motion images of 3% hydrogel; iv) stiffness measurements over a period of 13 d for 3%, 5%, and 10% acrylate‐functionalized dendritic polyglycerol‐based hydrogel fabricated in the presence of MCF‐7 cells.

Four‐arm PEG‐thiol was synthesized in accordance with the literature with a slight modification (Figure S3).^[^ ^25^ ^]^ It was functionalized with thiol group by using thiourea. The thiolation and hydrolysis were carried out at 80 °C after introducing a mesyl group. After purification, a pale yellowish precipitate was obtained in a high yield. The final product was characterized by ^1^H NMR and the functionalized thiol groups were quantified by the Ellman test, which showed ≈3.4 groups per molecule (Figure S6, Supporting Information). On the other hand, we functionalized dPG with acrylate via acrylation reaction using acryloyl chloride (**Figure** **4**i). After the dialysis purification, the dPG‐acrylate was obtained and kept as an aqueous stock solution. We quantified the acrylate groups according to the literature^[^ ^25^ ^]^ by ^1^H NMR end‐group analysis, showing roughly 5% functionalization or seven groups per molecule (Figure S2, Supporting Information).

The formation of the hydrogel involved simply mixing an aqueous solution of dPG‐acylate and four‐arm PEG‐thiol in phosphate buffer saline (PBS) (Figure 4i). In order to encapsulate the cells, we replaced PBS buffer with cell culture media (RPMI‐1640), and the cells were encapsulated in situ during gelation. Furthermore, we investigated to optimize the cell–cell and cell–gel interactions and so determine the recipe for the best hydrogel for tumor spheroid formation after encapsulation. In general, the viscoelasticity of hydrogels plays a crucial role in growing tumoroids, as the hydrogel must mimic the proper viscoelastic stiffness of natural ECM.^[11a,13]^ With appropriate stiffness and support from the hydrogel's 3D structure, cancer cells can expand to form tumor spheroids. As a result, we used the dilution approach to change the stiffness of our hydrogels from 10% to 5% to 3% (w/v) (Table S1, Supporting Information). To further evaluate rheological properties over time in the presence of MCF‐7 cells, we prepared in situ cell‐encapsulated hydrogel in a 48‐well plate with various gel concentrations, adding the cell culture media after complete gelation. The hydrogels’ stiffness was measured by rheometer at different times during incubation. The hydrogels were characterized by oscillatory shear on frequency sweep at a constant strain, resulting in a shear modulus graph that shows storage modulus (*G*ʹ) and loss modulus (*G*″). *G*ʹ represents the material's solid behavior, while *G*″ represents its liquid behavior. After the rheological measurement, the *G*ʹ and *G*″ values were plotted over the frequency range from 0.5 to 10 Hz, as shown in Figures S9–S11 (Supporting Information). The shear modulus graphs of the hydrogels at three different concentrations show that *G*ʹ is evidently predominant over *G*″, indicating that all gel samples have higher energy storage than energy dissipation. This proves the successful formation of a chemically crosslinked network in the hydrogels.

In order to simplify the shear modulus graphs while examining the degradability of the gels, we chose the *G*′ value at 1 Hz, which is directly related to the stiffness of the hydrogel. First, we compared the stiffness of three different gel concentrations at day 0, as seen in Figure 4ii. The 10% gel appears rigid and stiff, while the 5% gel is soft and elastic (Figure S8, Supporting Information), and the 3% hydrogel is the softest, behaving like slime (Figure 4iii). We determined the degradability of the hydrogel sample as it softened over time by comparing the hydrogel's stiffness at various incubation times. At 10% gel concentration, there was no discernible reduction in stiffness even after a prolonged incubation period (Figure 4iv). In contrast, from day 0 to day 13, the 5% and 3% hydrogel samples became progressively less stiff, showing a distinct decline of *G*′ in both cases: from 5.5 to 1.2 kPa for the 5% gel, and from 0.7 to 0.1 kPa for the 3% gel, respectively (Figure 4iv). Thus, it is evident that hydrogel samples at 5% and 3% show dynamic softening over time due to ester bond degradation of the acrylate group.

### 2.4 Multicellular Tumor Spheroid Formation from Immunomagnetically Separated MCF‐7 Cells and Anticancer Drug Screening

Following a detailed analysis of the chemical and rheological characteristics of hydrogels in relation to their effects on cell development, we found that the soft and slimy gels of 3% gel concentration permitted homogenous tumoroids to form, while no spheroid growth was seen with the 5% and 10% gels (Figure S12, Supporting Information). Soft hydrogels with viscoelastic, dynamically softening environments (from 0.7 to 0.1 kPa in stiffness measure) facilitated the formation of tumoroids. By contrast, stiffer gels constrained cell proliferation due to their rigid network and the resulting reduced mobility in their environment. We therefore chose 3% gel concentration as the ideal setting for MCTS formation in dPG‐acrylate based hydrogel.

**Figure 5:**
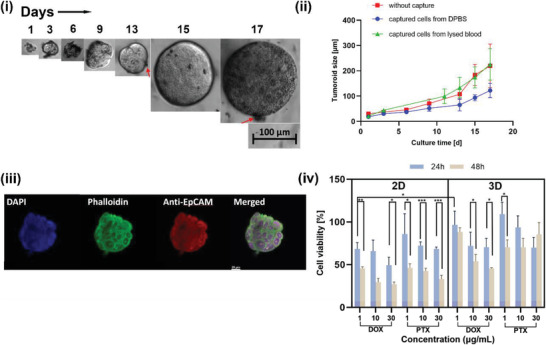
i) Bright‐field images illustrating the expansion of immunomagnetically isolated cancer cell into a 3D tumor spheroid when incubated in 3% hydrogel (dPG‐acrylate based). Scale bar = 100 µm; ii) comparative size change of 3D tumor spheroid from MCF‐7 cells captured from PBS or lysed blood over a span of 17 d with and without immunomagnetic isolation. Data are presented as mean ± standard deviation, *n* = 6; iii) confocal microscopy images of 3D tumor spheroids cultured from MCF‐7 cells captured in lysed blood for 15 d, stained with DAPI (nuclei), Phalloidin Fluoro594 (cellular cytoskeleton), and anti‐EpCAM Fluoro488 (EpCAM surface protein). Scale bar = 20 µm; iv) the dose‐dependent effects of the anticancer drugs doxorubicin (DOX) and paclitaxel (PTX) on the cultured tumor spheroids, with dPG‐based hydrogel as a growth matrix. The tumor spheroid growth from immunomagnetically isolated MCF‐7 cells within the hydrogel was tracked for 15 d prior to drug exposure. On day 15, the spheroids were exposed to three different drug concentrations ((i) 1, (ii) 10, (iii) 30 µg mL^−1^), and the effect of the drugs was studied for 24 and 48 h of exposure. *n* = 6, two sample *t*‐test, *p < 0.05, **p < 0.01, ***p < 0.001, mean ± SD.

After cells were homogenously combined and encapsulated in 3% gel, we observed the development of tumor spheroids under a microscope. MCF‐7 cells typically have a diameter of 19 µm,^[^ ^26^ ^]^ but under standard incubation conditions (37 °C, 5% CO_2_), the cells expanded to 70.71 µm on day 9 and 219.54 µm on day 17 of growth (Figure S14, Supporting Information). The tumoroids’ morphologies were consistent across different sites within the gel (Figures S12, Supporting Information), and Figure S15 (Supporting Information) shows a confocal laser scanning microscopy (CLSM) image of the structure of a single tumor spheroid. Comparing the culture times with the rheology measurements of the hydrogels, it is assumed that the expansion of single cells to spheroids can be attributed to the 3D support of the gel matrix. The chemistry and stiffness of the customized gel match the extracellular matrix (ECM) environment, facilitating 3D spheroid growth. In addition, the ester‐based hydrogel's degradation trend is consistent with tumor spheroids’ growth in size, since softer gels are expected to have loose network density, allowing easy expansion for tumor spheroids. To further substantiate the claim that cancer cells can be used for downstream analysis and that the 3D culture does not alter their fundamental characteristics, we used CLSM to examine EpCAM, which MCF‐7 cells overexpress. As seen in **Figure** **5**iii, the resulting imagery confirmed the presence of EpCAM protein on the tumoroid surface. EpCAM serves as a well‐established biomarker for cancer cells and their tumor initiating properties. In addition, it also serves as an adhesion molecule and as a promoter of cell proliferation. Therefore, EpCAM overexpression in 3D spheroids confirms their metastatic and stable proliferation nature. This is some of the most significant evidence for the viability and proliferation of EpCAM‐expressing cancer cells.

According to several described methods for tumoroid formation, e.g., the inverse drop method, magnetic levitation,^[^ ^27^ ^]^ tumoroids can form easily when a group of single cells aggregates together. However, tumoroid formation from a small number of cells is challenging due to the scarcity of CTCs isolated from blood samples. To prove that our gel matrix is viable with low seeding numbers of cells, we seeded 10, 100, 500, and 1000 cells to 100 µL of hydrogels (3%), observing the formation of tumor spheroids at each seeding concentration. Even when starting with just ten cells at the beginning of 3D culture in hydrogel, the tumor spheroid grew to 300 µm on day 13 of culture (Figure S16, Supporting Information). Our gel matrix validates the growth of tumoroids from a low number of cells while preserving the vital cell characteristics for further applications.

After tuning the hydrogel to optimize tumor spheroid growth, we also seeded immunomagnetically isolated cells in the optimized hydrogel candidates. In general, we observed a similar trend of growth in the presence of FeNP@dPG_anti‐EpCAM: single cells grew to 52.15 µm on day 9 and 122.33 µm on day 17 of culture (Figure 5i–iii). Explanations for the smaller sizes of these spheroids could be the restriction of iron nanoparticles and the slight change in hydrogel properties due to the presence of these particles. In spite of the downsizing, tumoroids still formed homogeneously throughout the hydrogels (Figure S13, Supporting Information). The hydrogels were found to be degraded after ≈15 d; this degradation not only coordinates the growth of tumoroids but also permits the grown tumoroids’ release.

The grown tumor spheroids were directly evaluated in drug screening experiments as candidates for downstream applications. We cultured cells in 2D well plates to serve as control in investigating the drugs’ effectiveness. Doxorubicin (DOX) and Paclitaxel (PTX) are the most commonly used anticancer drugs. We exposed 2D and 3D cultured MCF‐7 cells to DOX and PTX at different concentrations (1, 10, and 30 µg mL^−1^) for 24 and 48 h, then used the Celltiter‐Glo luminescence assay to determine cell viability. The results show that DOX has a better effect than PTX in general, leaving only 27.02% living cells after 48 h incubation at high concentration, i.e., 30 µg mL^−1^. Comparing the 2D and 3D models, an increased resistance to both drugs was clearly seen in 3D tumor spheroids (Figure 5iv). This observed behavior may be explained by the tumor developing cytostasis (a stage at which the drug treatment no longer causes cell death), which may have been facilitated by the hypoxic conditions present within the cultured MCTSs. These conditions are known to exist in in vivo solid tumors, contributing to drug resistance mechanisms.^[^ ^28^ ^]^ The densely packed tumor spheroids exhibit considerable resistance, making it difficult for drug molecules to penetrate them and demonstrating that the tumor microenvironment significantly affects the drug screening process. As a result, 3D tumor models that imitate ECM are significantly more effective than 2D models at simulating in vivo tumors and would help us understand underlying multidrug resistance mechanism, a crucial hurdle in the development of anticancer drugs, especially against CTCs.

## 3 Conclusion

Here, we have presented a straightforward and effective method for the capture and 3D growth of CTCs. A dual‐component polyglycerol‐based biomatrix was developed to immunomagnetically isolate CTCs with high purity and specificity and to further expand these cells into multicellular tumor spheroids. The first component consists of magnetic iron nanoparticles fabricated with an additional bio‐inert antifouling dPG coating and subsequently conjugated to anti‐EpCAM antibodies in order to extract CTCs. The designed FeNP@dPG_anti‐EpCAM particles displayed a capture efficiency of 93.6% for the MCF‐7 cell line; these cells were further used for expansion into MCTSs.

Furthermore, the immunomagnetically separated captured cells were directly encapsulated into a hydrogel, which was composed of dendritic polyglycerol and four‐arm polyethylene glycol, crosslinked by thiol‐Michael click reaction. We tuned the stiffness of the hydrogels and found that 3% gel concentration gave a perfectly suitable consistency for MCF‐7 cells to grow into MCTSs, which could then be easily extracted after the complete degradation of the gel. This behavior results from degradable ester bonds within the gel matrix, which lead to the dynamic softening of hydrogels over time (from 0.7 kPa at day 0 to 0.1 kPa at day 13). These rheological characteristics substantially encourage the growth of tumor spheroids of up to 300 µm in size. Moreover, the immunomagnetic separation followed by immediate 3D culture did not impede tumor development. By establishing the presence of the surface marker EpCAM, which the MCF‐7 cell line overexpressed, we further demonstrated that the growing MCTSs retain the cancer cell characteristics. We also anticipated that MCTSs can offer a wealth of therapeutically pertinent data for planning and researching drug resistance mechanisms and the effects of anti‐cancer medications on individual patients. We therefore explored the impact of generic anticancer drugs on MCTSs. The drug screening results showed higher apparent drug resistance in the 3D model than in 2D culture, showing that tumor spheroids can provide critical insights in future drug testing.

Our technique can connect liquid biopsy, oncological research, and cancer treatment, providing the next step for personalized therapeutic management.

## Conflict of Interest

The authors declare no conflict of interest.

## Supporting information

Supporting Information

## Data Availability

The data that support the findings of this study are available in the supplementary material of this article.
