# Supplementary material for: Polyglycerol‐Based Biomedical Matrix for Immunomagnetic Circulating Tumor Cell Isolation and Their Expansion into Tumor Spheroids for Drug Screening
Source: Adv Healthc Mater. 2023 Jul 21;12(26):2300842. doi: 10.1002/adhm.202300842 (PMC11469197; doi:10.1002/adhm.202300842)
Supplement: Supplementary file 1 — Supporting Information [file ADHM-12-2300842-s001.pdf]

ADVANCED HEALTHCARE MATERIALS

Supporting Information

for *Adv. Healthcare Mater.*, DOI 10.1002/adhm.202300842

Polyglycerol-Based Biomedical Matrix for Immunomagnetic Circulating Tumor Cell Isolation and Their Expansion into Tumor Spheroids for Drug Screening

Peng Tang, Boonya Thongrom, Smriti Arora and Rainer Haag**

Supplementary Information

Polyglycerol-based biomedical matrix for immunomagnetic circulating tumor cell isolation and their expansion into tumor spheroids for drug screening

Peng Tang, Boonya Thongrom, Smriti Arora, Rainer Haag**

Institute for Chemistry and Biochemistry, Freie Universität Berlin, Takustr. 3, Berlin, 14195, Germany.

Email: haag@chemie.fu-berlin.de, smriti@zedat.fu-berlin.de

Experimental Part

Materials and Methods:

Chemicals and solvents are HPLC grade, purchased from Merck (Steinheim, Germany) and used directly without any purification unless stated otherwise. Dialysis was performed with benzoylated dialysis tubes (width: 32 mm, molecular weight cut-off 2000 g/mol) purchased from Merck (Steinheim, Germany). Diethyl ether (100%) was purchased from VWR chemicals. N, N-Dimethylformamide (99.8%) was purchased from Acros Organics. Potassium hydroxide, DCM (99%) were purchased from Thermo-Fischer Scientific. 4-arm PEG 10 kDa was purchased from JenKem Technology USA Inc. Tris(2-carboxyethyl)phosphine hydrochloride was purchased from TCI Deutschland GmbH. Invitrogen™ Fixative-Free Lysing Solution, High-Yield Lyse buffer was purchased from ThermoFischer Scientific. The absorption spectra and photoluminescence (PL) spectra were measured on a PerkinElmer Lambda 25 UV–vis absorption spectrophotometer and an Edinburgh F900 fluorescent spectrometer equipped with a xenon arc lamp, respectively. NMR spectra were measured on a Jeol ECX 400 or Jeol ECP 500 MHz and 100 MHz spectrometer. The determination of thiol group was performed by an Agilent Cary 8454 UV-visible spectrophotometer using disposable semi-micro UV-cuvette. Dendritic polyglycerol (dPG) with average weight molecular weight of 10 kDa was prepared as previously reported[1] using an enhanced approach.[2]

Synthesis of dPG-succinic acid (SA)

Dendritic polyglycerol (1 g, 0.01 mmol, 10KDa) was dissolved in pyridine (10 mL) followed by the addition of succinic anhydride (2 g, 2 mmol). The reaction mixture was stirred at room temperature for 18 h before the pyridine was removed under vacuum at 40 °C. The remaining solid was dissolved in CH_2Cl_2 (50 mL) and washed with cold 0.2 N HCl (100 mL, 3X) or until the aqueous phase remained at pH 1. The organic phase was evaporated, and the transparent gel was collected (95% yield) for further reaction. In this case, dPG acrylate contains 60% acrylate or an estimated 81 groups per dPG molecule.

Synthesis of FeNPs

Synthesis of iron-oleate complex[3]:

A mixture of iron chloride (10.8g, 40mmol), and sodium oleate (36.5g, 120mmol) was dissolved in ethanol (80 ml), distilled water (60 ml), and hexane (140 ml). The resulting solution was heated to 60 °C for 4 hours under a nitrogen atmosphere. Next, the mixture was cooled down to room temperature and the organic phase was separated and washed three times with deionized water. Finally, solvents were evaporated in rotavapor to give a wax-like compound.

Synthesis of magnetic nanoparticles[3]:

Iron oleate (1g, 1.1 mmol), oleic acid (0.3g, 1 mmol), and 5.15g of 1-octadecene was heated to 320 °C for 1 hour under an inert atmosphere. Then the mixture was cooled to room temperature and washed several times with ethanol: acetone (1:1, v/v) as precipitating agent, and subsequently centrifuged. Finally, the nanoparticles wee resuspended in toluene. The synthesized particles were visualized under TEM.

Coating of magnetic nanoparticles with dopamine: The hydrophobic ligands of synthesized magnetic nanoparticles were replaced with dopamine molecules via ligand exchange method with some modifications.[4] To the stock solution of magnetic nanoparticles (1.2g in 20 ml), 5 ml methanol was added, and the resultant was sonicated for 5 mins. Then the mixture was washed with toluene (3 ml) using magnet. Further, 1ml of 100 mg/ml dopamine hydrochloride was added and the resultant was sonicated for ~15 mins which resulted in precipitation of particles. The precipitated particles were separated using magnet and washed with methanol (3X), MQ (3X) to remove any unreacted dopamine hydrochloride. Finally, the particles were redispersed in 80 ml MQ and stored as it is.

dPG coating of FeNPs (FeNP@dPG):

dPG carboxylic acid (0.12 g) was allowed to dissolve in DMF (24 mL) overnight. The following day, reaction temperature was maintained at 0℃, with addition of DIPEA (1.15 ml) and stirring for 30 mins. Then EDC (0.5 g), HOBt (0.3 g) were added to the reaction mixture and stirred for ~60 mins. Further, dopamine functionalized particles (1.2 g in 80 ml MQ) were added, and the reaction was allowed to stir overnight. Finally, the particles were washed with MQ (3X) with the

help of a magnet to remove any unreacted dPG-carboxylic acid. The particles were resuspended in MQ (8 mL). The synthesized particles were visualized under TEM.

Biofunctionalization of dPG coated FeNPs (FeNP@dPG_anti-EpCAM):

FeNP@dPG (30 mg in 2ml) were first activated in presence of EDC (127 mg), NHS (127 mg) for 3-4 hours in DMF (4 ml). After completion, nanoparticles were washed with PBS pH 7.4 (4 ml, 3X) and then redissolved in PBS pH 7.4 (4 ml). Further, the reaction was cooled to 0 °C, avidin (2 mg) was added, and the reaction was stirred overnight at 0 °C. Finally, the next day, particles were purified by washing with PBS pH 7.4 (2 ml, 2X) using a magnet, redispersed in PBS (2 ml) and kept in fridge until further use. The avidin functionalization was confirmed by checking its binding with Biotin-Atto 520. FeNP@dPG_avidin (3 mg in 0.2 ml) was diluted to 1 ml and further treated with Biotin-Atto 520 (0.01 mg in 0.01 ml) for 1 hour. After completion, nanoparticles were washed until no further colour change was observed. The nanoparticles were then diluted (1:20) and fluorescence measurement was recorded at λ_{ex} = 480 nm. The λ_{em} = 540 nm confirmed the avidin-biotin conjugation was confirmed.

Further, the biofunctionalization with anti-EpCAM antibody was performed by treating FeNP@dPG_avidin (5 mg in 2 ml) with Biotin-anti-EpCAM (0.01 mg in 0.01 ml) overnight at 4 °C. The particles were purified by washing with PBS pH 7.4 (2 ml, 2X) using a magnet, redispersed in PBS (2 ml) and kept in fridge until further use.

Synthesis of dPG-acrylate

To the DMF solution (30 mL) of 10 kDa dPG (3 g, 0.3 mmol, 1 eq.) under dry condition was added triethylamine (TEA, 0.5 mL, 3.6 mmol, 12 eq.) and the reaction mixture was cooled down with ice bath. Acryloyl chloride (0.24 mL, 3mmol, 10 eq.) was added dropwise to the reaction flask. The reaction was stirred for 1 d and afterward, it was subjected to purification by the dialysis using a 2 kDa cutoff benzoylated cellulose dialysis tube in water for 2 d. Then, the aqueous solution of dPG acrylate was collected, concentrated, and kept in the fridge (82% isolated yield). ^{1}H NMR (500 MHz, D_2O, δ (ppm)): 0.88 (3H, broad s, initiator backbone), 3.44 - 4.30 (m, backbone repeating units), 6.03 (1H, broad s), 6.25 (1H, broad s) and 6.45 – 6.48 (1H, broad s). The number of acrylate functional groups was calculated following the literature.[5] In

this case, dPG acrylate contains 5% acrylate or an estimated 7 groups per dPG molecule. **Figure S2**.

Synthesis of 4-arm PEG mesylate

10 kDa dried 4-arm PEG OH (**Figure S**, 7 g, 0.7 mmol, 1 eq.) was dissolved in anhydrous dichloromethane (DCM, 50 mL) and TEA (0.97 mL, 7 mmol, 10 eq.) was added to the reaction flask. The mixture was then cooled with an ice bath followed by dropwise addition of methanesulfonyl chloride (0.43 mL, 5.6 mmol, 8 eq.), and the reaction was then run for 1 d. Afterward, the crude product was washed thrice with brine, dried with Na_2SO_4, and later concentrated by a rotary evaporator. The crude mixture was precipitated in cooled diethyl ether, collected, and dried overnight under a vacuum. The precipitate product was obtained as a white powder with an 85% isolated yield. ^{1}H NMR (500 MHz, $CDCl_3$, δ (ppm)): 3.08 (3H, s), 3.40 - 3.78 (m), 4.37-4.38 (2H, t). **Figure S5**.

Synthesis of 4-arm PEG thiol

4-arm PEG mesylate (4.33 g, 0.43 mmol, 1 eq.) and thiourea (0.66 g, 8.7 mmol, 20 eq.) were added to the reaction flask followed by 1-propanol (10 mL). The reaction was carried at 80 °C for 1 d to obtain 4-arm PEG isothiouronium intermediate. After removing 1-propanol, KOH (0.024 g, 0.43 mmol, 4 eq.) and water (40 mL) were added to the reaction flask and the solution was then heated to 80 °C for 1 d. Afterward, tris(2-carboxyethyl)phosphine (TCEP, 0.5 g, 1.7 mmol, 4 eq.) was added to the crude mixture which was stirred for 2 h. The purification step is then explained, first saturating the crude mixture with NaCl, second extracting the product with DCM thrice and drying it with Na_2SO_4, third concentrating the DCM layer, and finally precipitating it in cooled diethyl ether. Dried 4-arm PEG thiol as a pale yellowish powder was obtained with a 90% isolated yield. ^{1}H NMR (700 MHz, $CDCl_3$, δ (ppm)): 1.59 (1H, t), 2.68-2.71 (2H, quat), 3.41 - 3.74 (m). 4-arm PEG thiol was also characterized by Ellman essay following the procedure from Thermofisher Scientific company. The number of thiol groups was quantified using the standard calibration curve of cysteine which contains 1 thiol group. The result shows that the number of thiol group on 4-arm PEG thiol is approximately 3.4 groups of thiols. **Figure S6**.

Rheological Analysis:

The rheological data of all hydrogel samples were characterized by Malvern Instruments Kinexus equipped with a cone plate of 20 mm diameter and 1 ° angle. The rheological test was conducted at 25 °C by an oscillatory frequency sweep strain-controlled where the constant strain is set at 1% at the frequency range of 0.1 - 100 Hz. The report stiffness value was directly related to the storage modulus at 1 Hz.

Transmission electron microscopy (TEM):

The samples were prepared by dripping the nanoparticle solution onto a Formvar/Carbon 200 mesh copper grid (Merck). Images were obtained from TEM mode of Hitachi SU8030 at high voltage of 15 kV, with working distance of 8.3 mm.

Cell Culture

Cell culture: Human breast cancer cell lines (MCF-7) and human cervical cancer cell lines (HeLa) were obtained from the American Type Culture Collection (ATCC). GFP^+-HeLa cells, derived from the HeLa cell line (ATCC; CCl-2), were transfected with and expression unit for a destabilized enhanced green fluorescent protein (d2EGFP, Clontech, Palo Alto, USA). Roswell Park Memorial Institute RPMI-1640 culture medium (Gibco) was supplemented with 10 % (v/v) fetal bovine serum (FBS) (Gibco) and 1 % (v/v) of penicillin-streptomycin solution (Gibco). All the cancer cell lines were routinely cultured at 37 ℃ with 5 % CO_2 in culture flasks, and were used from the same passage number for the same set of experiments.

Blood Lysis

Healthy human blood was purchased from German Red Cross (DRK Blutspendedienst Nord-Ost), and it was lysed as per the protocol using the lysing buffer (Invitrogen™ Fixative-Free Lysing Solution, High-Yield Lyse). The lysed blood was collected and utilized further for cancer cell capture experiments.

Cell Capture

Cancer cell capture: MCF-7 cells were pre-stained with CellTrace^{TM} violet dye (Thermo Fisher Scientific, Waltham, MA, USA) according to the standard protocol and the iron nanoparticles were stained by conjugating with biotin-dye (Sulfo-Cyanin-5-PEG3-Biotin, Lumiprobe GmbH, Germany). Stained cells were divided into 1mL Eppendorf tubes, then incubate together with the FeNPs@dPG_anti-EpCAM for 40 minutes in the incubator under mild rotation (rotation speed: 10 rpm). Then the mixture was put to a strong magnet while the supernatant was carefully removed by pipetting. The remains were suspended again with DBPS/human serum/lysed blood and then analysed with Attune NxT Flow Cytometer (Thermo Fisher Scientific, Waltham, MA, USA). Cells and particles were sorted by flow cytometer according to different selection markers. Captured cells appeared with both violet and red fluorescence while the uncaptured cells expressed only violet fluorescence. (**See Figure S7**).

The capture efficiency was calculated by the equation:

$$Capture\ efficiency\ (\%) = \frac{Captured\ cell\ number}{Total\ cell\ number\ counted\ by\ FACS} \times 100\%$$

Cells or tumoroids were fixed with 4 % paraformaldehyde at room temperature for 30 min, then washed with DPBS for 3 times. The cell membranes were permeabilized with 0.25% (v/v) Triton-X 100 in DPBS for 15 min, followed by washing 3 times with DPBS. Next, Samples were treated with DAPI and Phalloidin-iFluro594 reagent (Abcam, Cambridge, United Kingdom) for 30 min, then washed with DPBS. Fluorescence images were acquired on a Zeiss Axio Observer Z1 microscope or a Leica SP8 confocal microscope.

Statistical Analysis:

All tests were performed in at least three independent sessions. The quantified data are expressed as mean ± standard deviation. GraphPad Prism software was used for statistical analysis in this study. Differences between several groups were analyzed using one-way ANOVA with the Tukey multiple comparison test, whereas difference between the two groups was analyzed through two-tailed unpaired t-tests. In the quantitative images, the differences with a probability value (p) < 0.05 were considered statistically significant, and ns indicated no statistical significance.

All the results are reported as mean ± SD. The differences among groups were determined using one-way ANOVA analysis and student's t-test; *p < 0.05, **p < 0.01, ***p < 0.001, ****p < 0.0001.

Immunofluorescence staining:

Cells or tumoroids were fixed with 4 % paraformaldehyde at room temperature for 30 min, then washed with DPBS for 3 times. The cell membranes were permeabilized with 0.25% (v/v) Triton-X 100 in DPBS for 15 min, followed by incubating with 1 % (w/v) bovine serum albumin (BSA) in PBST (0.1 % v/v Triton-X 100 in DPBS) to prevent non-specific antibody binding. Next, samples were incubated with dye-labelled primary antibodies(anti-EpCAM Fluoro488) overnight at 4 ℃, then washed with twice with PBST and three times with DPBS. Afterwards, samples were stained with DAPI for 60 minutes in room temperature, followed by washing with PBST and DPBS. Immunofluorescence images were obtained using Leica SP8 confocal microscope.

Tumor Spheroid formation:

Cancer cells were prepared as suspension in RPMI-1640 medium at concentration of 2 x 10^5 cells/ml. Hydrogels with volume of 200 µL were made in 48-well plates. After all the gel components were added and well-mixed (Table S1), 50 µL of cell suspension (containing 10^4 cells) was to be encapsulated into one hydrogel by mixing again with all the gel components. After 3 hours, the hydrogel networks were thoroughly formed. Then 300 uL of RPMI-1640 medium was added to each well with hydrogels. The hydrogels were incubated at 37 ℃ with 5 % CO_2 in the 48-well plate, medium was changed every second day. During the cell growth, bright field images of cells were obtained by Zeiss Axio Observer Z1 microscope.

Different number cell seeding was done in a similar way in 96 well plate (with 10/100/500/1000 cells seeding in hydrogel with total volume of 100 µm).

Drug screening and cell viability test

Grown tumoroids and 2D cells were treated with doxorubicin hydrochloride (DOX, Sigma-Aldrich, 98% purity), paclitaxel (PTX, Thermo-Fischer Scientific) and their combination (DOX:PTX = 1:1) at concentration of 1 µg/mL, 10 µg/mL and 30 µg/mL, respectively. After 24 h and 48 h of drug treatment, the viability of cells was performed with CellTiter-Glo® luminescent

cell viability assay according to provided protocol. Luminescence signals were measured by Spark® microplate reader (TECAN, Männedorf, Switzerland).

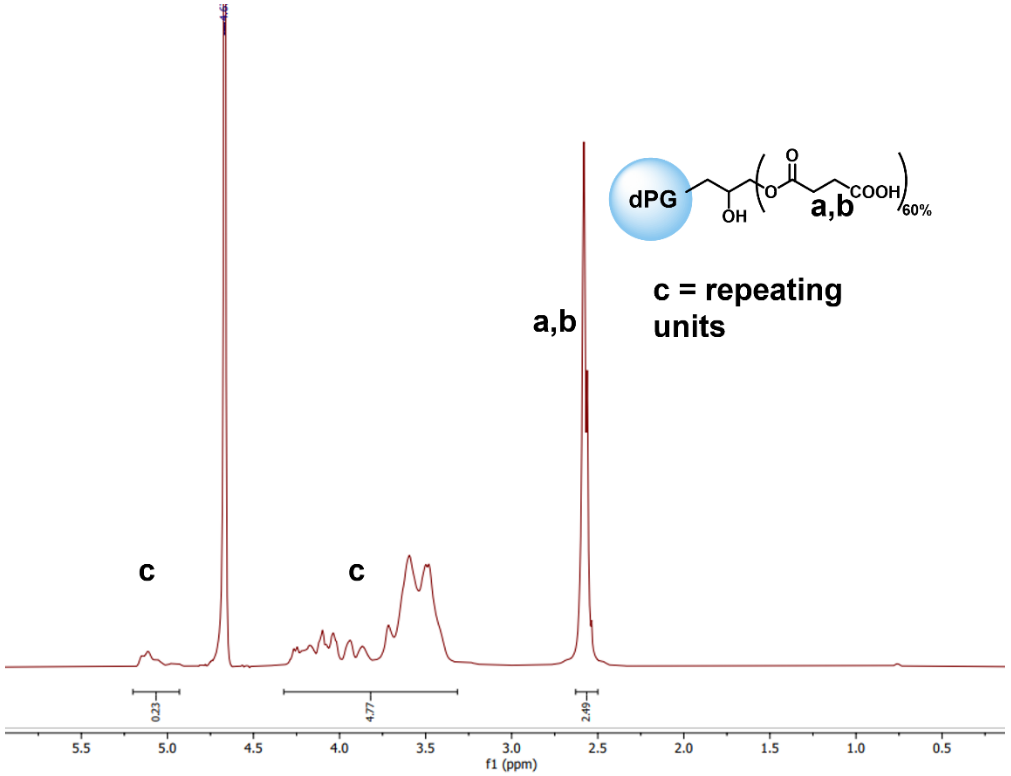

Figure S1. ^{1}H NMR (500 MHz, D_2O, δ (ppm)) of dPG-SA (dPG-succinic acid; 60% functionalized).

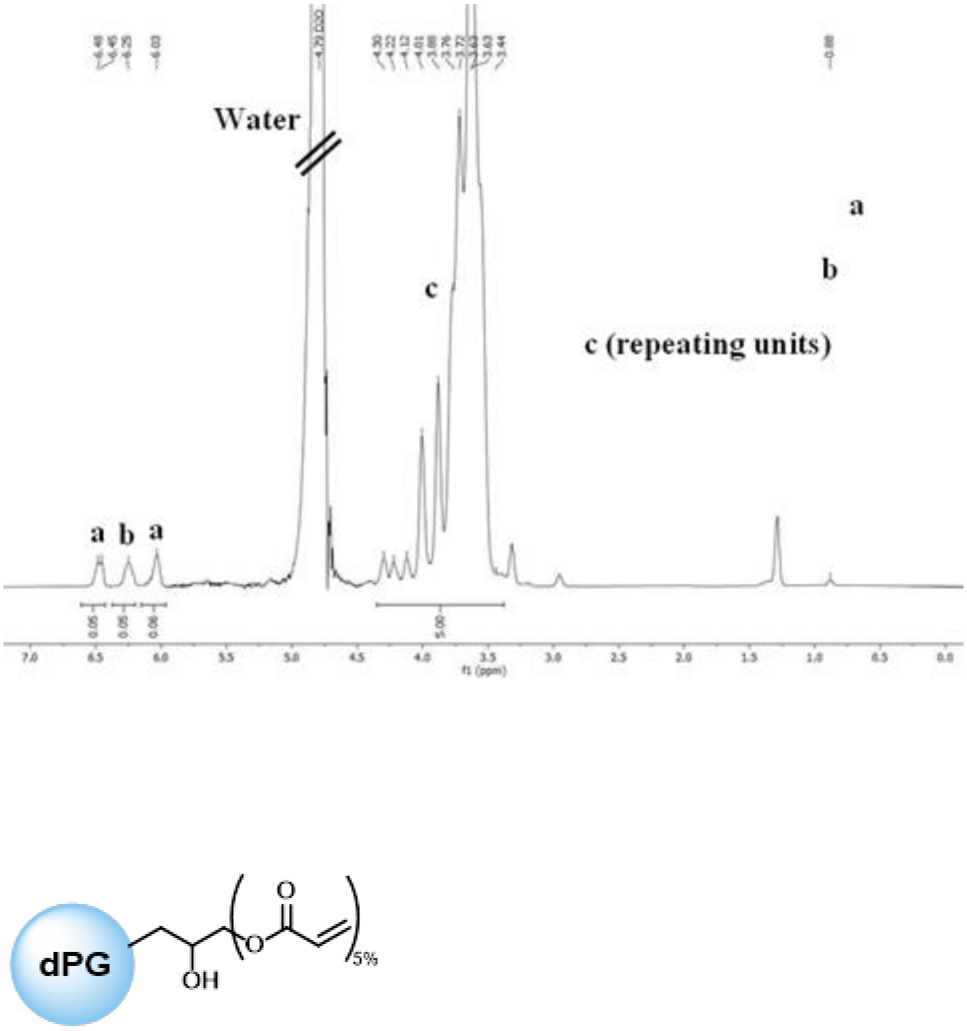

Figure S2. ^{1}H NMR (500 MHz, D_2O, δ (ppm)) of dPG-acrylate (5% functionalized).

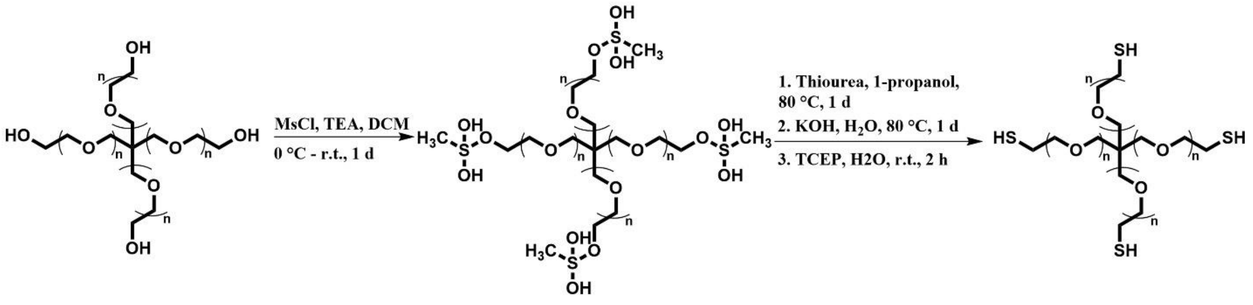

Figure S3. Synthesis of 4-arm PEG-thiol.

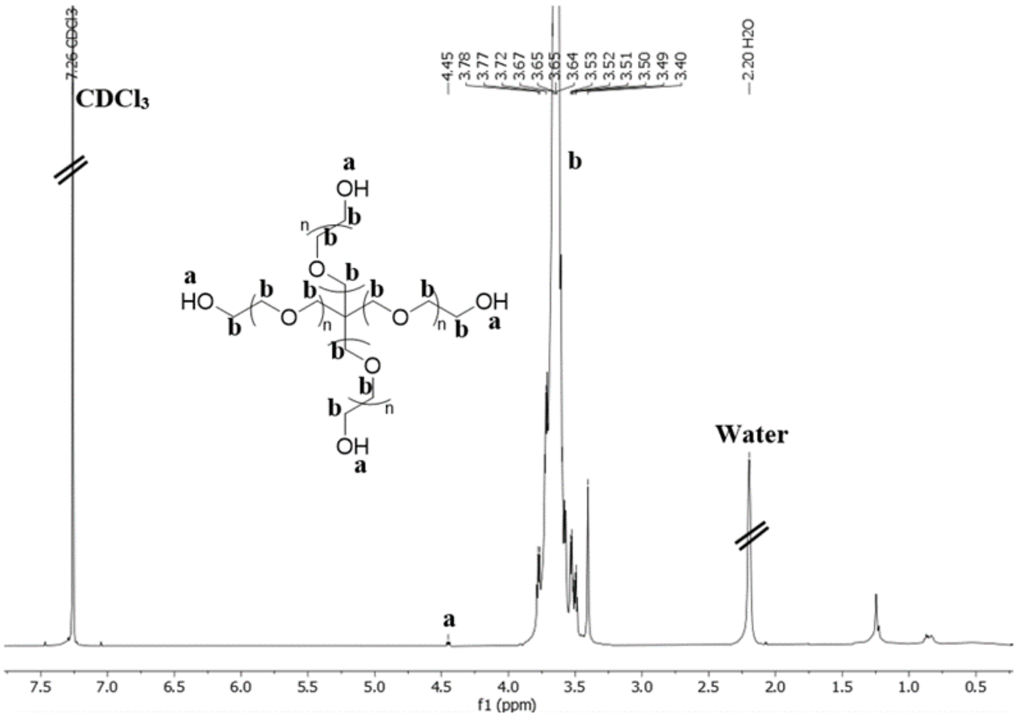

Figure S4. ^{1}H NMR (500 MHz, $CDCl_3$, δ (ppm)) of 4-arm PEG OH.

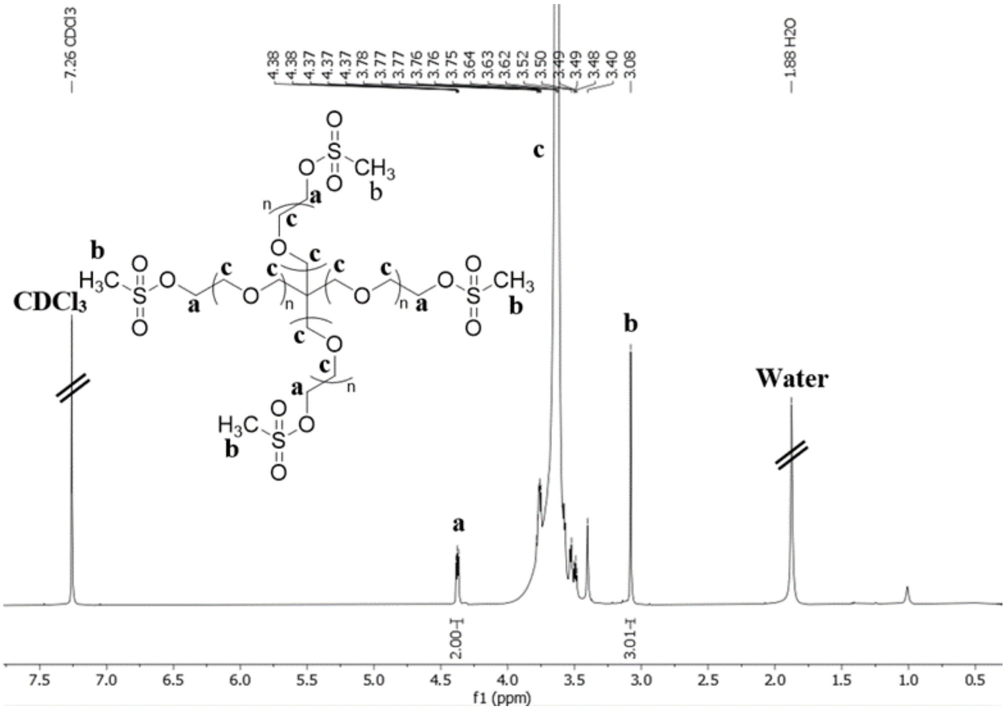

Figure S5. ^{1}H NMR (500 MHz, $CDCl_3$, δ (ppm)) of 4-arm PEG mesylate.

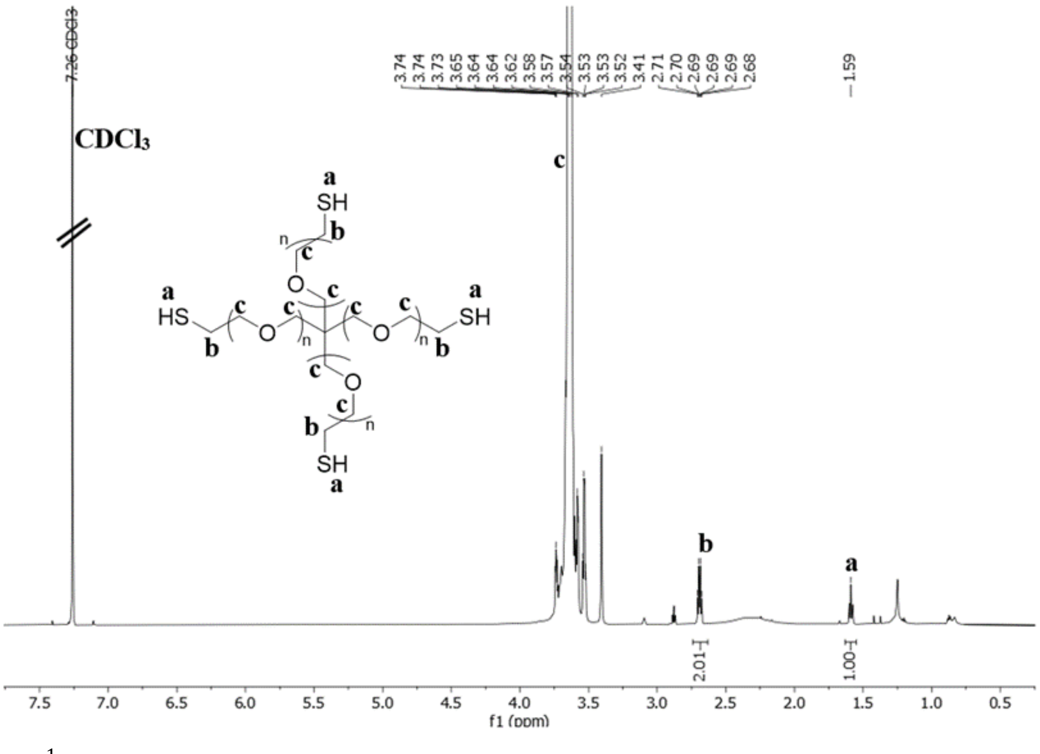

Figure S6. ^{1}H NMR (700 MHz, $CDCl_3$, δ (ppm)) of 4-arm PEG thiol.

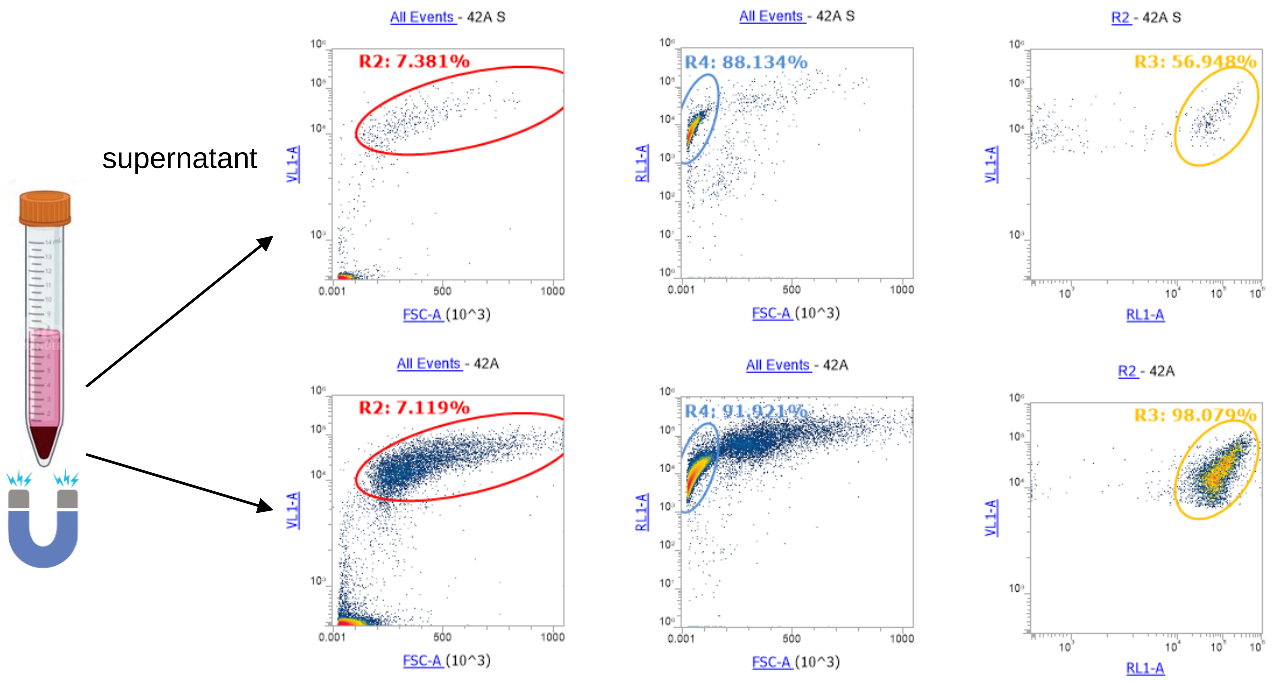

Figure S7. Representative overview of the flow cytometer analysis of immunomagnetically isolated MCF-7 cells. FeNPs were stained with red dye, MCF-7 cells were stained with violet dye, and gates were defined to progressively refine the selection. Top panel: depicts the supernatant population, Bottom panel: depicts the cells isolated after immunomagnetic isolation. R3 gate indicates the captured cells using FeNP@dPG_anti-EpCAM.

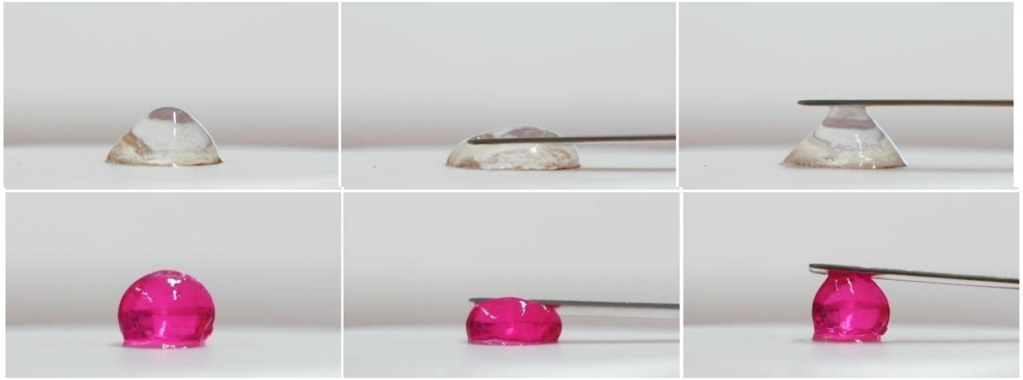

Figure S8. Motion images of = 3% gel (colorless) and 5% gel (in pink).

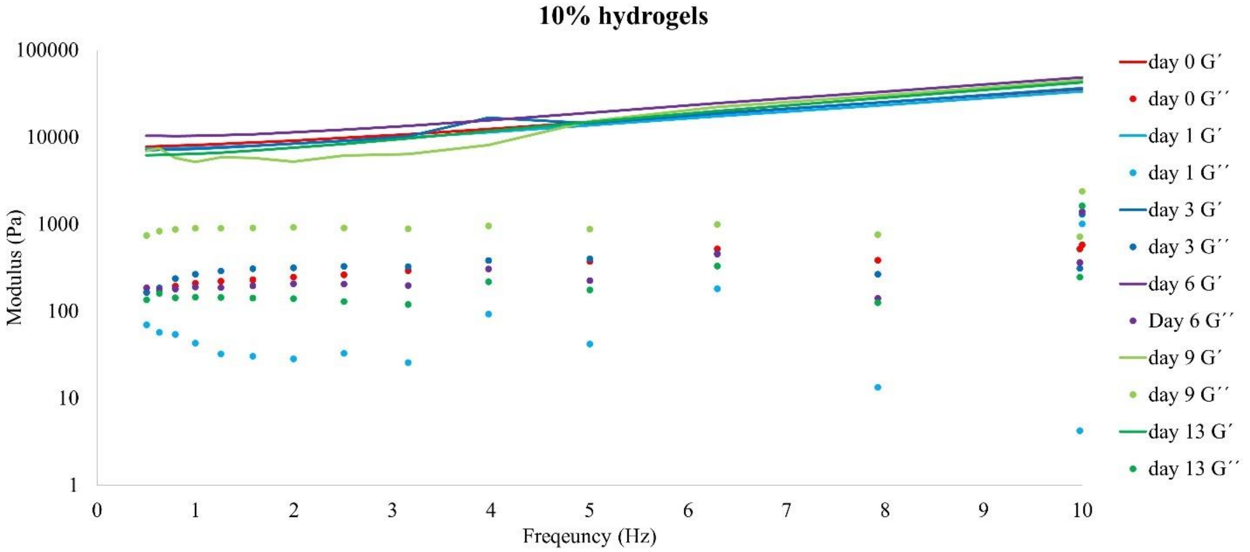

Figure S9. Storage (G´) and loss (G″) moduli as a function of frequency (Hz) for the 10% gel concentration.

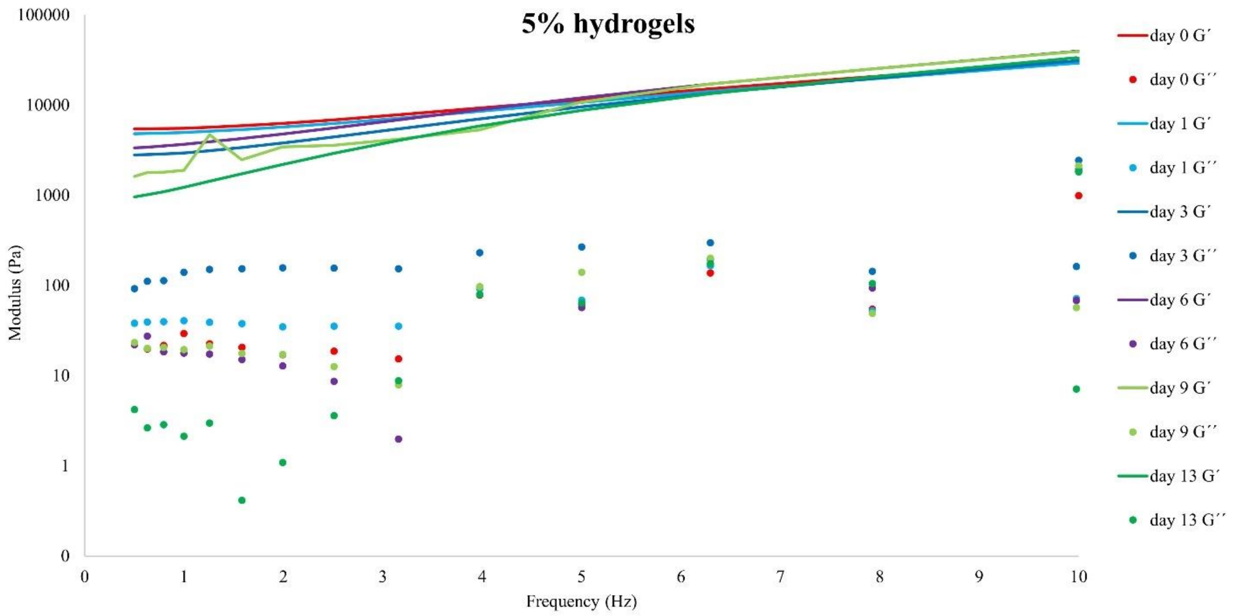

Figure S10. Storage (G´) and loss (G″) moduli as a function of frequency (Hz) for the 5% gel concentration.

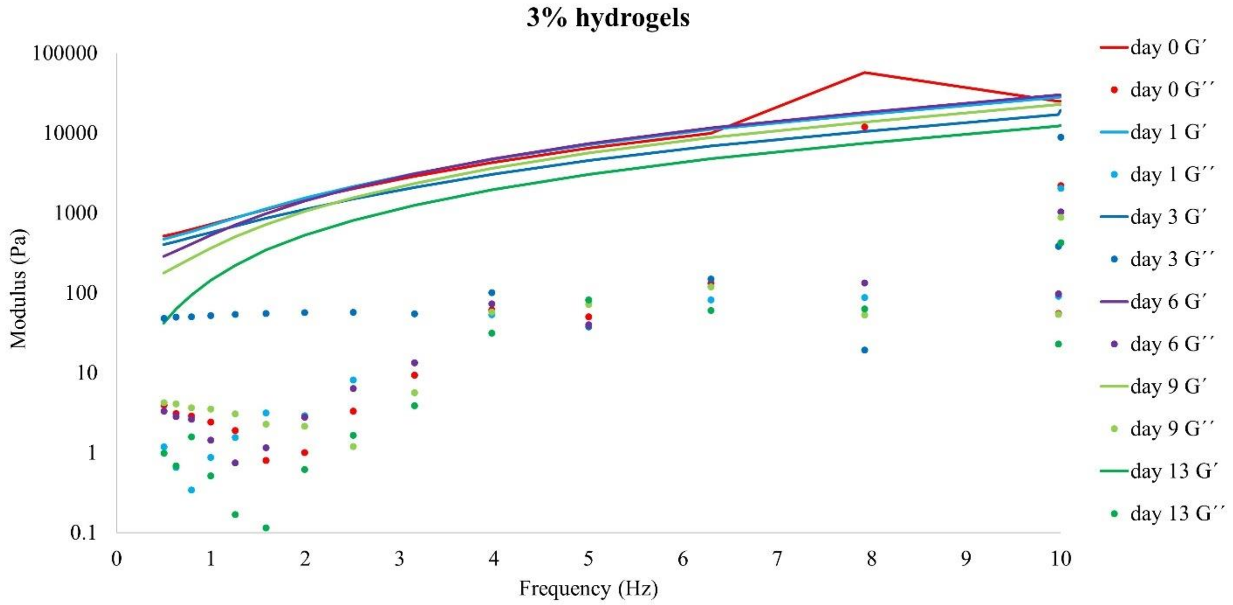

Figure S11. Storage (G´) and loss (G″) moduli as a function of frequency (Hz) for the 3% gel concentration

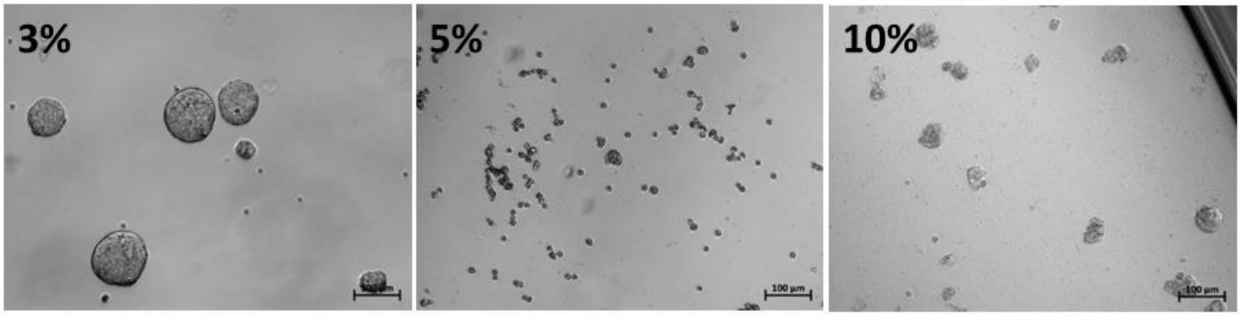

Figure S12. The tumoroid growth in different hydrogel concentrations (3, 5, 10 %).

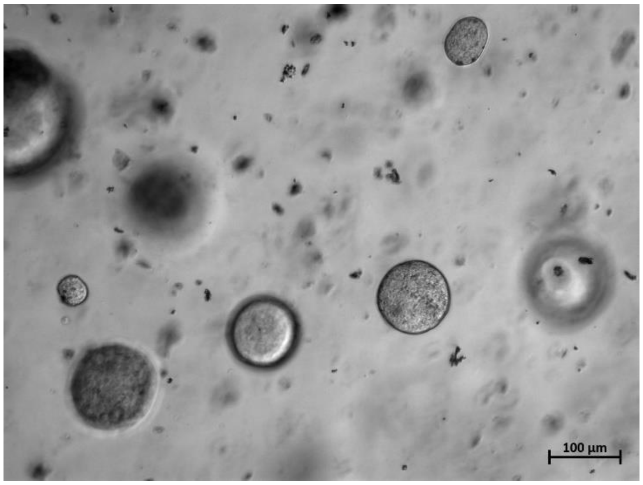

Figure S13. Brightfield image illustrating the expansion of MCF-7 cells (after immunomagnetic isolation) into 3D tumoroid when incubated in 3% hydrogel (dPG-acrylate based). The image depicts the expansion of tumor spheroids in different planes of hydrogel.

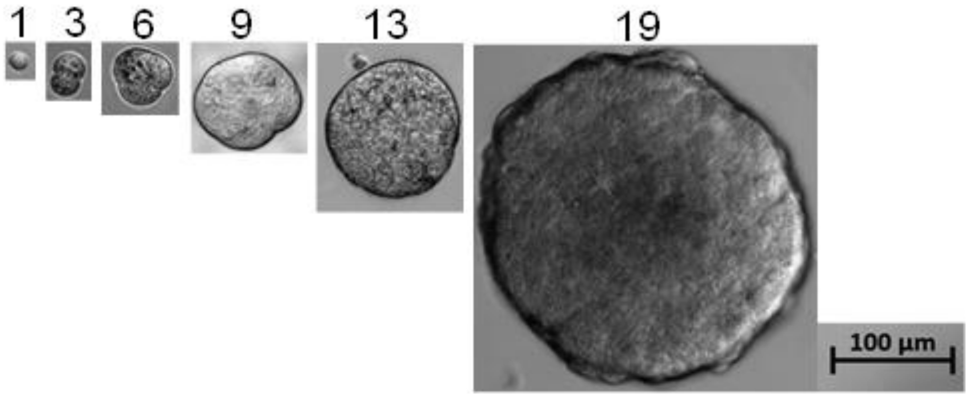

Figure S14. Brightfield images illustrating the expansion of MCF-7 cells (without immunomagnetic isolation) into 3D tumoroid when incubated in 3% hydrogel (dPG-acrylate based)

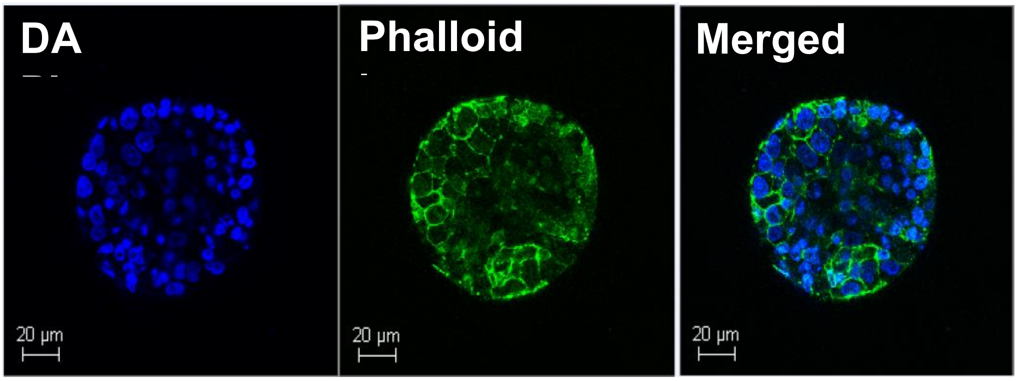

Figure S15. Confocal microscopy images of 3D tumoroid cultured for 15 days, stained with DAPI (nuclei), Phalloidin Fluoro594 (cellular cytoskeleton).

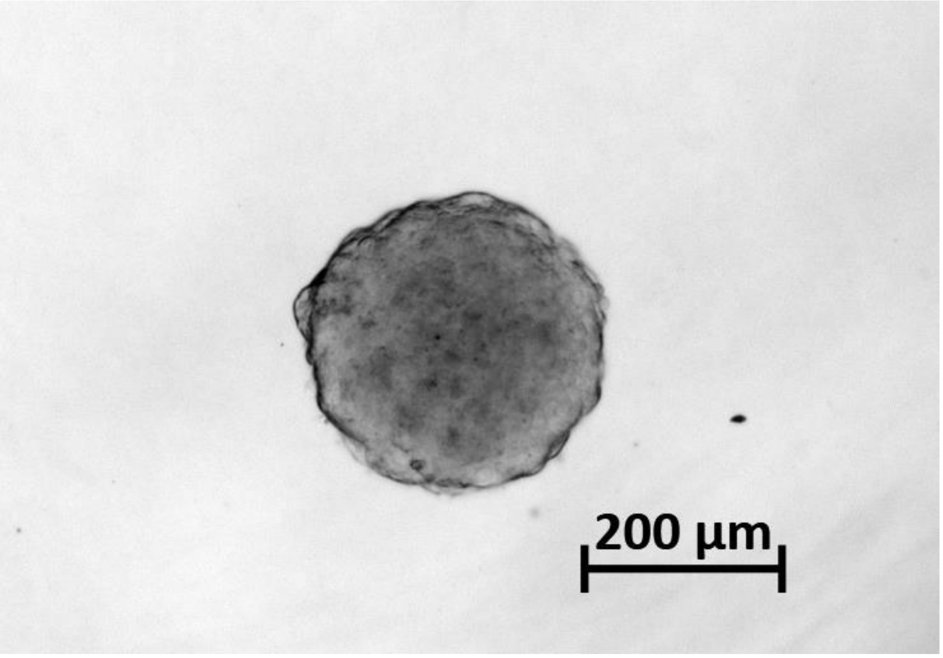

Figure S16. MCTS grown from 10 MCF-7 cells seeded within the hydrogel.

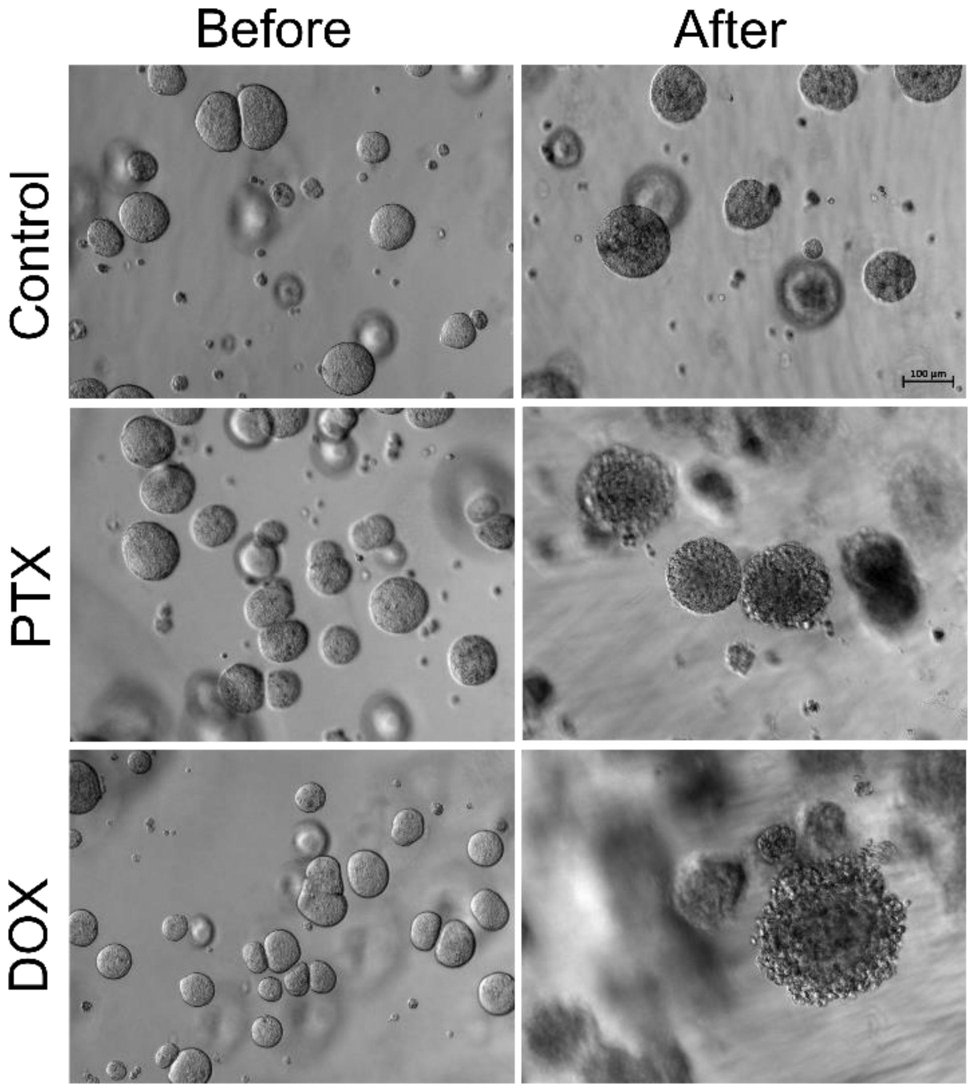

Figure S17. The morphological changes in grown tumoroids grown using the polyglycerol-based hydrogel on impact of different anticancer drugs, paclitaxel (PTX), doxorubicin (DOX).

Table S1. Preparation of the MCF-7 encapsulated *in situ* hydrogel formation at different gel concentration and different numbers of cells encapsulated.

No.	PEG(10% w/v)		dPG(24.5% w/v)		MCF-7*	PBS	Total	Conc.
	Volume (µL)	Mol (µmol)	Volume (µL)	Mol (µmol)	Amount (cells)	volume (µL)	volume (µL)	(%w/v)
1	55	0.55	18.5	0.45	3000	16.5	100	10
2	27.7	0.28	9.3	0.23	3000	53	100	5
3	16.7	0.17	5.7	0.14	3000	67.6	100	3
4	16.7	0.17	5.7	0.14	1000	67.6	100	3
5	16.7	0.17	5.7	0.14	500	67.6	100	3
6	16.7	0.17	5.7	0.14	100	67.6	100	3
7	16.7	0.17	5.7	0.14	10	67.6	100	3

*The volume of MCF-7 solution added is always 10 µL.

Table S2. Dose dependent effect of different drugs on grown 3D tumoroids in comparison to control hydrogels (with no drug treatment) in dPG based hydrogel over an incubation time of 24 h and 48 h.

Drugs	Dosage (µg/mL)	% Live cells	
		24 h post-drug treatment	48 h post-drug treatment
**Doxorubicin**	1	96.71 ± 0.16	88.25 ± 0.05
	10	71.94 ± 0.16	54.01 ± 0.08
	30	70.50 ± 0.10	45.46 ± 0.01
**Paclitaxel**	1	109.17 ± 0.14	70.28 ± 0.09
	10	93.51 ± 0.14	70.23 ± 0.11
	30	69.88 ± 0.12	85.58 ± 0.14

References

[1] aA. Sunder, R. Hanselmann, H. Frey, R. Mülhaupt, *Macromolecules* **1999**, *32*, 4240-4246; bR. Haag, A. Sunder, J.-F. Stumbé, *Journal of the American Chemical Society* **2000**, *122*, 2954-2955.

[2] M. Wallert, J. Plaschke, M. Dimde, V. Ahmadi, S. Block, R. Haag, *Macromolecular Materials and Engineering* **2021**, *306*, 2000688.

[3] J. Park, K. An, Y. Hwang, J.-G. Park, H.-J. Noh, J.-Y. Kim, J.-H. Park, N.-M. Hwang, T. Hyeon, *Nature Materials* **2004**, *3*, 891-895.

[4] J. Sherwood, Y. Xu, K. Lovas, Y. Qin, Y. Bao, *Journal of Magnetism and Magnetic Materials* **2017**, *427*, 220-224.

[5] B. Thongrom, M. Dimde, U. Schedler, R. Haag, *Macromolecular Chemistry and Physics* **2023**, *224*, 2200271.
